# Correlation between log_e_ GDR and hyperuricemia in patients with type 2 diabetes mellitus: a cross-sectional study

**DOI:** 10.3389/fendo.2025.1637373

**Published:** 2025-10-01

**Authors:** Pei Liu, Baolan Ji, Yan Peng

**Affiliations:** ^1^ School of Clinical Medicine, Shandong Second Medical University, Weifang, Shandong, China; ^2^ Department of Endocrinology, Linyi People’s Hospital Affiliated to Shandong Second Medical University, Linyi, Shandong, China

**Keywords:** type 2 diabetes mellitus, hyperuricemia, insulin sensitivity, loge GDR, insulin resistance

## Abstract

**Introduction:**

Hyperuricemia (HUA), an important health concern, is closely associated with insulin sensitivity. The natural log transformation of the glucose disposal rate (log_e_ GDR) is a new model of insulin sensitivity in patients with type 2 diabetes mellitus (T2DM). The association between HUA and insulin resistance has been demonstrated by other insulin resistance indices. However, the correlation between log_e_ GDR and HUA has not been explored. This study explored the interaction between log_e_ GDR and HUA in patients with T2DM.

**Methods:**

This study involved 2,352 patients with T2DM. Biochemical and clinical data were collected. Morning blood samples were collected after an overnight fast for serum uric acid measurement. All the parameters required for log_e_ GDR calculation, including triglycerides, γ-glutamyl transferase, urinary albumin-to-creatinine ratio, and body mass index, were also collected. The correlation between the log_e_ GDR and HUA was analyzed.

**Results:**

Patients with HUA had lower log_e_ GDR values than those without (P< 0.001). HUA prevalence decreased significantly with increasing log_e_ GDR quartiles (P< 0.001). Multivariable regression analysis revealed that log_e_ GDR was independently associated with HUA (odds ratio: 0.279, 95% confidence interval: 0.170–0.459). Log_e_ GDR’s area under the receiver operating characteristic curve (0.706, 95%CI = 0.664-0.747) was superior to other indices.

**Discussion:**

Log_e_ GDR correlates strongly with HUA and demonstrates significant HUA predictive value in patients with T2DM.

## 1 Introduction

Uric acid is synthesized mainly in the liver, intestines, and vascular endothelium as the end product of an exogenous pool of purines, and endogenously from damaged, dying, and dead cells, whereby nucleic acids, adenine, and guanine are degraded into uric acid (1). Hyperuricemia (HUA), a metabolic syndrome (MetS) caused by disrupted purine metabolism (2), is characterized by a uric acid level of >420 µmol/L in men and >360 µmol/L in women (3). HUA is also an independent risk factor for the development of obesity, chronic kidney disease, hypertension, type 2 diabetes, dyslipidemia, coronary heart disease, and stroke (4). Numerous studies have shown that insulin resistance (IR) has a close physiological and pathological association with HUA (5). IR may contribute to HUA (6), and reducing IR may reduce serum uric acid (SUA) levels and the risk of gout (7, 8). HUA can interfere with insulin signaling and decrease endothelial nitric oxide availability (9), which is considered the primary factor that couples endothelial dysfunction with IR (10). An animal experimental study in Japan found that insulin can promote uric acid reabsorption through urate transporter 1 and ATP-binding cassette subfamily G member 2 (11). Furthermore, HUA and insulin sensitivity are associated with MetS. People with MetS may experience HUA because of IR, fatty liver, and dyslipidemia (12, 13).

However, IR is clinically challenging to identify. Because of its high cost and technical complexity, the hyperinsulinemic euglycemic clamp, which is considered the gold standard for IR identification (14), is not routinely employed in clinical practice. Therefore, many alternative IR indicators based on anthropometric and biochemical parameters have been proposed. Ciardullo et al. recently proposed the natural log transformation of the glucose disposal rate (log_e_ GDR) as an innovative model of IS prediction in individuals with type 2 diabetes mellitus (T2DM). Log_e_ GDR includes common clinical parameters: triglycerides (TG), urinary albumin-to-creatinine ratio (UACR), γ-glutamyl transferase (GGT), and body mass index (BMI), which reflect lipid metabolism, renal function, hepatic function, and body weight-related metabolic risk. They are critical components of HUA pathogenesis and key biomarkers of MetS. MetS and IR are closely associated with HUA. Therefore, as a comprehensive surrogate IS index, we hypothesize that log_e_ GDR may be strongly associated with HUA. Moreover, no studies have confirmed the association between log_e_ GDR and HUA. So this study explored the interaction between log_e_ GDR and HUA in patients with T2DM. This study aimed to evaluate whether loge GDR is independently associated with hyperuricemia in patients with T2DM and to compare its predictive performance with other insulin resistance indices.

## 2 Materials and methods

Our study involved inpatients with T2DM (age: 18–87 years) at the Department of Endocrinology, Linyi People’s Hospital, from January 2020 to March 2023. Exclusion criteria: (1) incomplete basic clinical data or unclear medical history and (2) comorbidities, including severe infections involving other systems, malignancy, or major organ failure. The HUA group had 336 cases (uric acid: >420 and >360 µmol/L in men and women, respectively), and the non-HUA group had 2016 cases.

Moreover, our analysis included other commonly used indicators of IR indices as covariates, including homeostatic model assessment of insulin resistance (HOMA-IR), triglyceride glucose index (TyG index), triglyceride glucose-body mass index (TyG-BMI), triglyceride/high-density cholesterol-lipoprotein ratio (TG/HDL-c ratio), triglyceride-glucose and gamma-glutamyl transferase (TYG-GGT), triglyceride-glucose-alanine aminotransferase (TyG–ALT), the single-point insulin sensitivity estimator (SPISE), metabolic score for IR (METS-IR), improved triglyceride glucose index (TyGIS), and estimated glucose disposal rate (eGDR_BMI_). This is because literature indicates a strong positive connection between the other commonly used indicators of IR and HUA among adults. Consequently, we incorporated these markers into our analysis.

### 2.1 Anthropometric and biochemical measurements

We recorded patient demographics and clinical characteristics, including age, sex, duration of diabetes, height, weight, smoking habit, and alcohol consumption. Blood pressure was measured in duplicate using a validated electronic sphygmomanometer (recording systolic and diastolic blood pressure [SBP/DBP]) after resting in a seated position for ≥5 min in a quiet, temperature-controlled environment. Fasting blood samples were collected in the morning and analyzed for TG, total cholesterol (TC), high-density lipoprotein-cholesterol (HDL-c), low-density lipoprotein-cholesterol (LDL-c), aspartate aminotransferase (AST), alanine aminotransferase (ALT), GGT, fasting blood glucose (FBG), glycosylated hemoglobin (HbA1c, high-performance liquid chromatography), uric acid, and hemoglobin (Hb) using a biochemical autoanalyzer (Cobas c 702, Roche, Germany). UACR was measured using an autoanalyzer (Beckman Coulter AU5821). Fasting serum insulin (FINS) was measured using direct chemiluminescence on a fully automated system (Aptio Automation, Siemens, USA). Bioelectrical impedance analysis (Omron DUALSCAN HDS-2000, Kyoto, Japan) was used to assess visceral fat area (VFA) and subcutaneous fat area (SFA).

1. Parameter calculations $\text{BMI}=\frac{\text{weight (kg)}}{{\text{height (m)}}^{2}}$ (15).
2.
$\text{TyG index}=\ln\left[\frac{\text{TG (mg/dL)}\times \text{FBG (mg/dL)}}{2}\right]$ (16).
3.
TyG-BMI=TyG×BMI (17).
4.
TyG-GGT=TyG×GGT (18).
5.
$\text{TyG–ALT index}=\ln\left(\frac{\text{fasting TG [mg/dL]}\times \text{fasting glucose [mg/dL]}\times \text{ALT [IU/L]}}{2}\right)$ (19).
6.
$\text{TG/HDL-c ratio}=\frac{\text{TG (mmol/L)}}{\text{HDL-c (mmol/L)}}$ (20).
7.
$\text{HOMA-IR}=\frac{\text{FBG (mmol/L)}\times \text{FINS (mIU/L)}}{22.5}$ (21).
8.
$\text{SPISE index}=\frac{600\times {\text{HDL-c [mg/dL]}}^{0.185}}{{\text{TG [mg/dL]}}^{0.2}\times {\text{BMI [kg/m}}^{2{]}^{1.338}}}$ (22).
9.
$\begin{array}{c} TyGIS=A\times TyG+B\times BodyWeight\left(kg\right)+\\ C\times FastingInsulin\left(pmol/L\right)+D\times LBM\left(\%\right)+\\ E,A=-0.4670326,B=-0.1219702,C=-0.0226746,D=0.2214735,E=-9.7092789 \end{array}$ LBM=0.296×weight (kg)+41.813×height (m)−43.293 (23).
10.
$\text{METS-IR}=\frac{\ln\left[2\times \text{FPG (mg/dL)}+\text{TG (mg/dL)}\right]\times \text{BMI}}{\ln\left[\text{HDL-c (mg/dL)}\right]}$ (24).
11.
$eGFR=175\times \text{Scr}{\left(\text{mg}/\text{dL}\right)}^{-1.234}\times {\text{age}}^{-0.179}\times \{\begin{array}{ll} 0.79 & \text{female}\\ 1.00 & \text{man} \end{array}$ (25).
12.
$\begin{array}{c} \log_{e}\text{GDR}=5.3505-0.3697\times \log_{e}\left(\text{GGT, IU/L}\right)-0.2591\times \log_{e}\left(\text{TG, mg/dL}\right)-\\ \;\;\;\;\;\;0.1169\times \log_{e}\text{(UACR, mg/g})-\left(0.0279\times {\text{BMI, kg/m}}^{2}\right) \end{array}$ (26).
13.
eGDRBMI= 19.02−(0.22×BMI)− (3.26×HT)− (0.61×HbA1c) (BMI = body $\begin{array}{c} \text{mass index }\left(\text{kg}/\text{m}2\right),\text{ HT }=\text{ hypertension }\\ \left(\text{yes }=\;1/\text{no }=\;0\right),\text{and HbA}1\text{c }=\text{ HbA}1\text{c }\left(\%\right)) \end{array}$ (27).

### 2.2 Statistical analyses

Statistical analyses were conducted using SPSS version 26.0 (SPSS Inc., Chicago, IL, USA). Normally distributed continuous variables, non-normally distributed data, and categorical variables were presented as mean ± standard deviation (SD), median (interquartile range), and frequencies (%). Differences between two groups of normally or non-normally distributed data were compared using independent sample t-tests or Mann–Whitney U tests, respectively. Differences between four or more groups were compared using one-way analysis of variance (ANOVA) for normally distributed data or Kruskal–Wallis tests for non-normally distributed data, with *post-hoc* multiple comparisons being performed using Student–Newman–Keuls tests where applicable. Chi-square tests were used for all categorical variable comparisons. Logistic regression analysis was used to assess independent HUA correlates. Receiver operating characteristic (ROC) curve analysis was used to evaluate the ability of log_e_ GDR to predict HUA. All statistical tests were two-tailed, with p< 0.05 indicating statistically significant differences.

## 3 Results

### 3.1 Baseline clinical and biochemical characteristics

The patients’ clinical and biochemical profiles are presented in **Table 1**. This study enrolled 2,352 patients (mean age: 57.3 ± 13.2 years). Sex was not significantly different between the two groups (men: 41.3% vs. 41.1%, p > 0.05). Compared with the non-HUA group (n=2,016), age, HDL-c, eGFR, Hb, SPISE, TyGIS, eGDR_BMI,_ and log_e_ GDR were significantly lower in the HUA group (n=336), but BMI, VFA, SFA, TG, FBG, FINS, ALT, AST, GGT, UACR, TyG index, TyG-BMI, TyG-GGT, TyG-ALT, TG/HDL-c ratio, HOMA-IR, and METS-IR were significantly higher (all p< 0.05). Smoking (%), drinking (%), SBP, DBP, TC, LDL-C, duration of diabetes, and HbA1c levels were not significantly different between the two groups (all p > 0.05).

**Table 1:** Clinical and biochemical characteristics by presence of HUA.

Variables	All	Non-HUA	HUA group;	P value
Number	2352	2016	336	
Sex (male, n, %)	971 (41.3%)	833 (41.3%)	138 (41.1%)	0.932
Smoking (n, %)	372(15.8%)	313 (15.5%)	59 (17.6%)	0.348
Drinking (n, %)	319(13.6%)	270 (13.4%)	49 (14.6%)	0.560
Age (years)	57.3 ± 13.2	58.0 ± 12.3	53.3 ± 16.8	<0.001
Duration of diabetes (years)	8.7(2.0-13.0)	8.6(2.0-13.0)	9.2(2.0-15.0)	0.099
BMI (kg/m2)	25.39 ± 3.88	25.17 ± 3.71	26.73 ± 4.59	<0.001
VFA (cm2)	92.00 (63.25-119.00)	90.45(63.00-117.00)	102.28(71.50-131.00)	<0.001
SFA (cm2)	186.98 (138.00-228.00)	183.08(136.00-223.00)	212.85 (56.00-262.50)	<0.001
SBP (mmHg)	129.73 ± 19.21	129.69 ± 19.17	130.00 ± 19.48	0.782
DBP (mmHg)	80.30 ± 11.74	80.27 ± 11.48	80.48 ± 13.24	0.789
TC (mmol/L)	4.85 ± 1.32	4.84 ± 1.28	4.90 ± 1.49	0.500
LDL-c (mmol/L)	3.04 ± 1.10	3.05 ± 1.07	3.00 ± 1.28	0.534
TG (mmol/l)	1.91 (0.99-2.09)	1.83(0.94-1.98)	2.35(1.29-2.77)	<0.001
HDL-c (mmol/L)	1.18 ± 0.35	1.20 ± 0.35	1.07 ± 0.32	<0.001
FBG (mmol/L)	9.18 ± 4.02	9.08 ± 3.87	9.78 ± 4.81	0.011
FINS (μU/mL)	19.91 (10.37-22.81)	19.54(10.30 ± 22.34)	22.25 (10.86-27.37)	0.009
HbA1c (%)	9.41 ± 2.28	9.45 ± 2.26	9.21 ± 2.39	0.090
ALT (U/L)	23.89 (12.88-26.33)	22.84(13.00-26.05)	30.17 (12.00-30.60)	0.005
AST (U/L)	21.33 (14.00-22.70)	20.57(14.00-22.10)	25.88 (14.00-28.38)	<0.001
GGT (U/L)	31.19 (15.00-32.00)	30.23(15.00-30.00)	39.96(17.00-41.98)	<0.001
eGFR (mL/min/1.73 m^2^)	119.33 ± 37.16	123.26 ± 34.36	95.53 ± 44.02	<0.001
UACR (mg/g)	219.91 (6.10-46.78)	179.78(6.10-35.68)	460.74 (6.80-190.75)	<0.001
Hb (g/L)	138.86 ± 18.81	139.47 ± 18.36	135.21 ± 20.97	0.001
TyG index	9.22 ± 0.80	9.17 ± 0.79	9.51 ± 0.81	<0.001
TyG-BMI	234.89 ± 46.07	231.50 ± 43.66	255.03 ± 54.33	<0.001
TYG-GGT	279.81(135.33-305.26)	266.76(132.02-290.11)	357.71 (153.56-411.69)	<0.001
TyG–ALT	12.16 ± 1.09	12.09 ± 1.05	12.53 ± 1.24	<0.001
TG/HDL-c ratio	1.84 (0.78-2.03)	1.71(0.74-1.89)	2.62 (1.10-2.84)	<0.001
HOMA-IR	7.55 (3.43-9.71)	7.31(3.40-9.43)	9.09 (3.69-11.52)	0.045
SPISE	6.36 ± 1.87	6.49 ± 1.83	5.62 ± 1.90	<0.001
TyGIS	4.64 ± 2.00	4.75 ± 1.90	3.95 ± 2.47	<0.001
METS-IR	41.57 ± 8.93	40.84 ± 8.36	45.92 ± 10.76	<0.001
Log_e_ GDR	1.86 ± 0.43	1.90 ± 0.42	1.60 ± 0.45	<0.001
eGDR_BMI_	1.86 ± 0.43	6.65 ± 2.13	6.21 ± 2.09	0.001

Normally distributed variables were expressed as mean ± standard deviation (SD), and intergroup comparisons were conducted using independent two-sample t-tests. Abnormally distributed variables were presented as median (25th percentile~75th percentile), and comparisons between the two groups were made using the Mann–Whitney U test. Categorical variables were reported as percentages (%) and were compared by chi-square test. A two-sided P-value< 0.05 was considered statistically significant.

BMI, body mass index; VFA, visceral fat area; SFA, subcutaneous fat area; SBP, systolic blood pressure; DBP, diastolic blood pressure; TC, total cholesterol; LDL-c, low-density lipoprotein cholesterol; TG, triglyceride; HDL-c, high-density lipoprotein cholesterol; FBG, fasting blood glucose; FINS, fasting serum insulin; ALT, alanine aminotransferase; AST, aspartate aminotransferase; GGT, gamma-glutamyl transferase; eGFR, estimated glomerular filtration rate; UACR, urinary albumin to creatinine ratio; Hb, hemoglobin; TyG–ALT, triglyceride–glucose–alanine aminotransferase index; HOMA-IR, homeostatic model assessment of insulin resistance; SPISE, the single point insulin sensitivity estimator; TyGIS, improved triglyceride glucose index; METS-IR, metabolic score for IR; log_e_ GDR, a natural log transformation of the glucose disposal rate; eGDR_BMI_, estimated glucose disposal rate.

**Figure 1:**
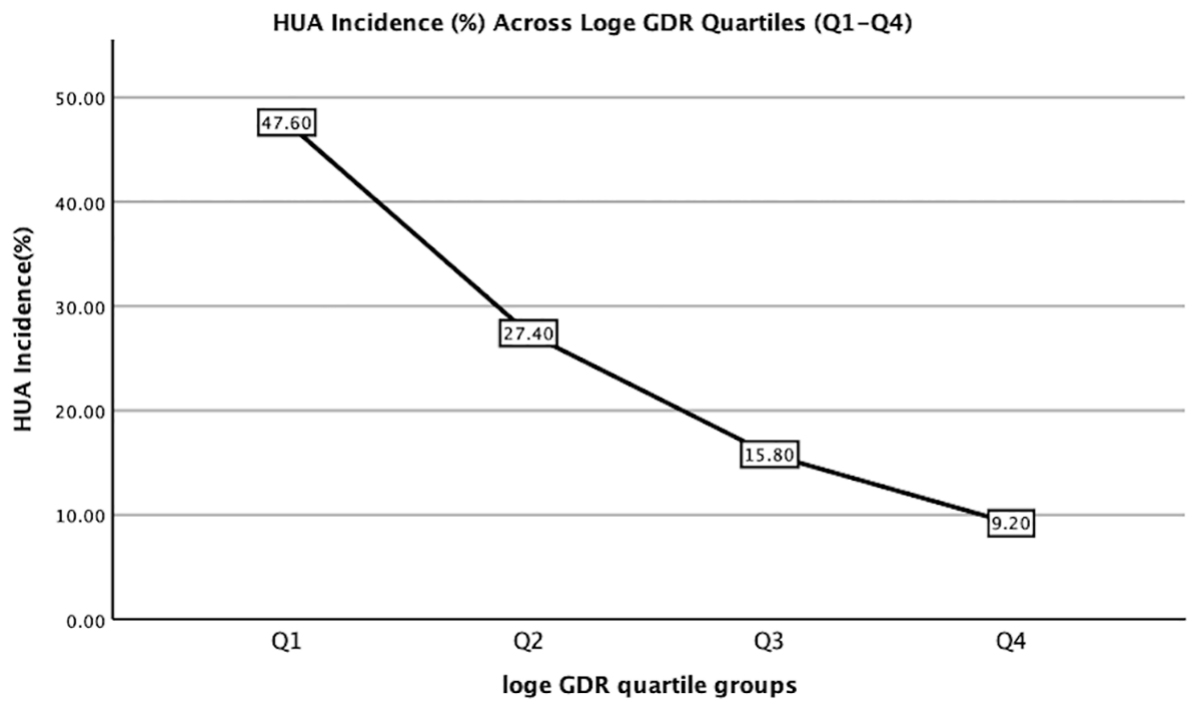
Hyperuricemia incidence (%) across Loge GDR quartiles (Q1:<1.59; Q2: 1.59–1.90; Q3: 1.90–2.17; Q4: ≥2.17). Trend test: P<0.001.

Participants were stratified into four groups based on log_e_ GDR quartiles (Q1–Q4; **Table 2**). The levels of HDL-c, eGFR, SPISE, and eGDR_BMI_ increased with increasing log_e_ GDR quartiles (all p< 0.001), whereas sex (male, %), smoking (%), drinking (%), age, BMI, VFA, SFA, SBP, DBP, TC, LDL-c, TG, FBG, FINS, HbA1c, ALT, AST, GGT, uric acid, UACR, Hb, TyG index, TyG-BMI, TyG-GGT, TyG-ALT, TG/HDL-c ratio, HOMA-IR, TyGIS, METS-IR, and HUA decreased significantly (all p< 0.001). The duration of diabetes did not differ between the four groups (p = 0.073).We further analyzed the relationship between the incidence of HUA and quartile groups of log_e_ GDR (**Figure 1**). The results demonstrated a significant inverse trend, with HUA incidence showing a progressive decline across increasing quartiles of log_e_ GDR (P for trend<0.001).

**Table 2:** Baseline characteristics across quartiles of Log_e_ GDR.

Variables	Q1 (0.25-1.58)	Q2 (1.59-1.89)	Q3 (1.90-2.16)	Q4 (2.17-3.12)	P value
Number	578	596	578	600	
Sex (male, n, %)	320(55.4%)	244(40.9%)	207(35.8%)	200(33.3%)	<0.001
Smoking (n, %)	134(23.2%)	100(16.8%)	79(13.7%)	59(9.8%)	<0.001
Drinking (n, %)	124(21.5%)	84(14.1%)	56(9.7%)	55(9.2%)	<0.001
Age (years)	53.90 ± 14.4	58.6 ± 12.8^a^	59.1 ± 12.1^a^	57.5 ± 12.6^a^	<0.001
Duration of diabetes (years)	8.5(2.0 - 14.0)	9.3(3.0 - 14.0)	8.2(2.0 - 12.0)	8.9(3.0 - 13.0)	0.073
BMI (kg/m2)	27.70 ± 4.28	26.05 ± 3.58^a^	24.86 ± 2.98^ab^	23.03 ± 2.96^abc^	<0.001
VFA (cm2)	118.06 (90.00 - 147.25)	100.67(76.00 - 124.00)^a^	86.35(64.00 - 109.00)^ab^	64.99(44.00 - 86.00)^abc^	<0.001
SFA (cm2)	224.95(170.00 - 271.25)	200.93(156.00 - 235.00)^a^	177.89(138.00 - 214.50)^ab^	147.22(108.00 - 184.00)^abc^	<0.001
SBP (mmHg)	134.90 ± 20.11	131.47 ± 19.27^a^	128.70 ± 17.64^ab^	124.03 ± 18.09^abc^	<0.001
DBP (mmHg)	84.17 ± 12.60	80.91 ± 11.80^a^	79.61 ± 10.57^a^	76.64 ± 10.68^abc^	<0.001
TC (mmol/L)	5.19 ± 1.55	4.99 ± 1.34^a^	4.76 ± 1.15^ab^	4.44 ± 1.06^abc^	<0.001
LDL-c (mmol/L)	3.13 ± 1.18	3.19 ± 1.16	3.06 ± 0.98	2.80 ± 1.06^abc^	<0.001
TG (mmol/l)	3.34(1.56 - 3.61)	1.92(1.25 - 2.38)^a^	1.42(1.04 - 1.66)^ab^	0.98(0.69 - 1.20)^abc^	<0.001
HDL-c (mmol/L)	1.05 ± 0.36	1.14 ± 0.31^a^	1.21 ± 0.32^ab^	1.33 ± 0.36^abc^	<0.001
FBG (mmol/L)	9.85 ± 3.79	9.72 ± 4.54	9.02 ± 3.57^ab^	8.16 ± 3.89^abc^	<0.001
FINS (μU/mL)	20.61(12.43 - 23.95)	20.85(12.65 - 23.64)	19.30(9.64 - 22.10)	18.95(6.23 - 20.76)	<0.001
HbA1c (%)	9.59 ± 2.13	9.60 ± 2.25	9.46 ± 2.31	9.01 ± 2.39^abc^	<0.001
ALT (U/L)	35.23(15.50 - 44.25)	23.10(13.30 - 26.75)^a^	20.17(12.50 - 23.80)^ab^	17.54(11.30 - 20.58)^abc^	<0.001
AST (U/L)	28.51(15.68 - 32.85)	20.41(14.20 - 21.88)^a^	19.05(13.48 - 20.40)^a^	17.56(13.00 - 20.00)^a^	<0.001
GGT (U/L)	61.76(27.00 - 63.58)	28.40(19.00 - 33.00)^a^	20.67(16.00 - 24.00)^ab^	14.66(11.00 - 17.00)^abc^	<0.001
UA (µmol/L)	346.74 ± 106.03	296.72 ± 91.05^a^	268.58 ± 86.73^ab^	249.91 ± 87.58^abc^	<0.001
eGFR (mL/min/1.73 m^2^)	110.75 ± 43.01	115.16 ± 40.14^a^	122.70 ± 32.11^ab^	127.85 ± 29.80^abc^	<0.001
UACR (mg/g)	637.80(12.45 - 456.35)	188.04(7.70 - 69.23)^a^	48.88(6.10 - 23.63)^ab^	13.77(4.20 - 11.10)^ab^	<0.001
Hb (g/L)	140.80 ± 21.72	138.20 ± 19.72	140.21 ± 16.58	136.34 ± 16.50	<0.001
HUA (n, %)	160(27.7%)	92(15.4%)^a^	53(9.2%)^ab^	31(5.2%)^abc^	<0.001
TyG index	9.79 ± 0.84	9.40 ± 0.68^a^	9.08 ± 0.56^ab^	8.60 ± 0.57^abc^	<0.001
TyG-BMI	271.75 ± 50.35	245.21 ± 37.76^a^	225.60 ± 28.51^ab^	198.07 ± 28.84^abc^	<0.001
TYG-GGT	551.09(265.80 - 626.01)	264.15(179.06 - 318.21)^a^	186.93(143.11 - 218.83)^ab^	125.72(95.65 - 45.31)^abc^	<0.001
TyG–ALT	13.07 ± 1.16	12.36 ± 0.86^a^	11.93 ± 0.75^ab^	11.32 ± 0.74^abc^	<0.001
TG/HDL-c ratio	3.45(1.47 - 3.83)	1.87(1.07 - 2.29)^a^	1.29(0.81 - 1.50)^ab^	0.80(0.49 - 0.98)^abc^	<0.001
HOMA-IR	8.82(4.73 - 11.40)	8.47(4.43 - 10.27)	6.87(3.24 - 9.31)	6.17(2.05 - 7.20)	<0.001
SPISE	5.00 ± 1.44	5.84 ± 1.41^a^	6.55 ± 1.32^ab^	8.02 ± 1.77^abc^	<0.001
TyGIS	3.93 ± 1.80	4.18 ± 1.96	4.83 ± 1.81^ab^	5.53 ± 2.03^abc^	<0.001
METS-IR	48.63 ± 10.03	43.31 ± 7.32^a^	39.74 ± 5.86^ab^	34.82 ± 5.45^abc^	<0.001
eGDR_BMI_	1.27 ± 0.26	1.75 ± 0.09	2.03 ± 0.08	2.37 ± 0.16	<0.001

Normally distributed variables were expressed as mean ± standard deviation (SD), and intergroup comparisons were conducted using one-way analysis of variance (ANOVA). Abnormally distributed variables were presented as median (25th percentile~75thpercentile), and we compared the four groups using the Kruskal–Wallis test. *Post hoc* multiple comparisons were performed using the Student–Newman–Keuls (SNK) test for pairwise group comparisons. Categorical variables were reported as percentages (n, %), with group differences assessed by chi-square test. A two-tailed P-value< 0.05 was considered statistically significant. ^a^ P<0.05 versus Q1;^b^ P<0.05 Q3、Q4 versus Q2;^c^ P<0.05 Q4 versus Q3.

BMI, body mass index; VFA, visceral fat area; SFA, subcutaneous fat area; SBP, systolic blood pressure; DBP, diastolic blood pressure; TC, total cholesterol; LDL-c, low-density lipoprotein cholesterol; TG, triglyceride; HDL-c, high-density lipoprotein cholesterol; FBG, fasting blood glucose; FINS, fasting serum insulin; ALT, alanine aminotransferase; AST, aspartate aminotransferase; GGT, gamma-glutamyl transferase; UA, uric acid; eGFR, estimated glomerular filtration rate; UACR, urinary albumin to creatinine ratio; Hb, hemoglobin; TyG–ALT, triglyceride–glucose–alanine aminotransferase index; HOMA-IR, homeostatic model assessment of insulin resistance; SPISE, the single point insulin sensitivity estimator; TyGIS, improved triglyceride glucose index; METS-IR, metabolic score for IR, eGDR_BMI_, estimated glucose disposal rate.

### 3.2 Univariate regression analysis

Univariate logistic regression analysis was used to identify factors that may be associated with HUA (**Table 3**). This analysis revealed that BMI, VFA, SFA, TG, FBG, AST, ALT, GGT, UACR, TyG index, TyG-BMI, TyG-GGT, TyG-ALT, TG/HDL-c ratio, HOMA-IR, and METS-IR correlated positively with HUA, whereas age, HDL-c, eGFR, Hb, SPISE, TyGIS, eGDR_BMI_, and log_e_ GDR were negatively correlated (all p< 0.05). Sex (male, %), smoking (%), drinking (%), duration of diabetes, SBP, DBP, TC, LDL-c, FINS, and HbAIc did not correlate with HUA (all p > 0.05).

**Table 3:** Univariate regression analysis for HUA.

Variables	OR (95% CI)	P value
Sex (male)	1.010(0.799-1.278)	0.932
Smoking	1.158(0.853-1.552)	0.348
Drinking	1.103(0.794-1.532)	0.560
Age	0.974(0.966-0.983)	<0.001
Duration of diabetes	1.011(0.994-1.028)	0.194
BMI (kg/m2)	1.102(1.071-1.134)	<0.001
VFA (cm2)	1.102(1.071-1.134)	<0.001
SFA (cm2)	1.005(1.004-1.007)	<0.001
SBP (mmHg)	1.001(0.995-1.007)	0.782
DBP (mmHg)	1.001(0.992-1.011)	0.767
TC (mmol/L)	1.034(0.948-1.128)	0.452
LDL-c (mmol/L)	0.962(0.865-1.070)	0.479
TG (mmol/l)	1.042(1.008-1.079)	0.017
HDL-c (mmol/L)	0.238(0.157-0.361)	<0.001
FBG (mmol/L)	1.039(1.013-1.067)	0.003
FINS (μU/mL)	1.003(0.998-1.008)	0.207
HbA1c (%)	0.955(0.906-1.007)	0.091
ALT (U/L)	1.010(1.006-1.014)	<0.001
AST (U/L)	1.015(1.009-1.021)	<0.001
GGT (U/L)	1.002(1.000-1.004)	0.022
eGFR (mL/min/1.73 m^2^)	0.976(0.972-0.980)	<0.001
UACR (mg/g)	1.000(1.000-1.000)	<0.001
Hb (g/L)	0.988(0.983-0.994)	<0.001
TyG index	1.673(1.454-1.926)	<0.001
TyG-BMI	1.010(1.008-1.013)	<0.001
TYG-GGT	1.001(1.000-1.001)	<0.001
TyG–ALT	1.421(1.282-1.576)	<0.001
TG/HDL-c ratio	1.120(1.076-1.166)	<0.001
HOMA-IR	1.039(1.017-1.061)	<0.001
SPISE	0.744(0.690-0.802)	<0.001
TyGIS	0.844(0.788-0.904)	<0.001
METS-IR	1.060(1.047-1.074)	<0.001
Log_e_ GDR	0.215(0.164-0.282)	<0.001
eGDR_BMI_	0.909(0.860-0.961)	0.001

A univariate regression analysis was conducted to identify the factors associated with hyperuricemia.

BMI, body mass index; VFA, visceral fat area; SFA, subcutaneous fat area; SBP, systolic blood pressure; DBP, diastolic blood pressure; TC, total cholesterol; LDL-c, low-density lipoprotein cholesterol; TG, triglyceride; HDL-c, high-density lipoprotein cholesterol; FBG, fasting blood glucose; FINS, fasting serum insulin; ALT, alanine aminotransferase; AST, aspartate aminotransferase; GGT, gamma-glutamyl transferase; eGFR, estimated glomerular filtration rate; UACR, urinary albumin to creatinine ratio; Hb, hemoglobin; TyG–ALT, triglyceride–glucose–alanine aminotransferase index; HOMA-IR, homeostatic model assessment of insulin resistance; SPISE, the single point insulin sensitivity estimator; TyGIS, improved triglyceride glucose index; METS-IR, metabolic score for IR; log_e_ GDR, a natural log transformation of the glucose disposal rate, eGDR_BMI_, estimated glucose disposal rate.

### 3.3 Multivariable regression analysis

Multivariable regression analysis of independent association between age, BMI, VFA, SFA, TG, HDL-c, FBG, FINS, ALT, AST, GGT, eGFR, UACR, Hb, TyG index, TyG-BMI, TYG-GGT, TyG-ALT, TG/HDL-c ratio, HOMA-IR, SPISE, TyGIS, METS-IR, eGDR_BMI_, and log_e_ GDR revealed that log_e_ GDR (odds ratio [OR]: 0.279, 95% confidence interval [CI]: 0.170–0.459), age (OR: 0.946, 95% CI: 0.930–0.963), AST (OR: 1.013, 95% CI: 1.002–1.023), UACR (OR: 1.000, 95% CI: 1.000–1.000), Hb (OR: 0.981, 95% CI: 0.970–0.992), and eGFR (OR: 0.971, 95% CI: 0.964–0.979) were independently associated with HUA (**Table 4**).

**Table 4:** The independent variables for HUA.

Variables	B	SE	Wald	P	OR	95.0% CI for OR
Age	−0.055	0.009	39.612	<0.001	0.946	0.930 - 0.963
AST	0.012	0.005	5.870	0.015	1.013	1.002 - 1.023
eGFR	−0.029	0.004	55.882	<0.001	0.971	0.964 - 0.979
UACR	0.000	0.000	4.547	0.033	1.000	1.000 - 1.000
Hb	−0.019	0.006	10.781	0.001	0.981	0.970 - 0.992
Log_e_ GDR	−1.276	0.254	26.332	<0.001	0.279	0.170 - 0.459

The independent variables for hyperuricemia was assessed by logistic regression analysis.

AST, aspartate aminotransferase; eGFR, estimated glomerular filtration rate; UACR, urinary albumin to creatinine ratio; Hb, hemoglobin; Log_e_ GDR, a natural log transformation of the glucose disposal rate; CI, confidence interval; OR, odd ratio; SE, standard error.

### 3.4 Area under the ROC curve analysis

A comparison of the predictive performance of log_e_ GDR with its components (BMI, GGT, UACR, and TG), the aforementioned IR indices (TG/HDL-c ratio, TyG index, TyG-GGT, TyG-BMI, TyG-ALT, HOMA-IR, SPISE, TyGIS, METS-IR, and eGDR_BMI_), HUA-related common markers (TC, HDL-c, and LDL-c), and regression model variables (age, AST, eGFR, and Hb) revealed that Log_e_ GDR had a superior predictive ability (AUC = 0.706, **Table 5**). Furthermore, we performed pairwise comparisons of the areas under the ROC curves using the paired-sample design feature in ROC analysis within SPSS version 26. Differential ROC analysis showed that log_e_ GDR was higher than the TG/HDL-c ratio, TG, SPISE, METS-IR, TyG index, TyG-BMI, HDL-c, TyG-ALT, TyG-GGT, UACR, GGT, BMI, TyGIS, age, AST, Hb, HOMA-IR, TC, LDL-c, and eGDR_BMI_ (all p< 0.05). However, the difference between log_e_ GDR and eGFR was not significant (p = 0.936).

**Table 5:** Analysis of the areas under the ROC curves for predicting HUA.

Variables	AUC	SE	95.0% CI
eGFR	0.708	0.024	0.664 - 0.755
Log_e_ GDR	0.706	0.021	0.664 - 0.747
TG/HDL-c ratio	0.667	0.022	0.624 - 0.710
TG	0.659	0.022	0.616 - 0.701
SPISE	0.644	0.024	0.592 - 0.691
METS-IR	0.632	0.024	0.585 - 0.680
TyG index	0.631	0.022	0.588 - 0.674
TyG-BMI	0.629	0.024	0.581 - 0.676
HDL-c	0.618	0.022	0.574 - 0.662
TyG–ALT	0.616	0.024	0.569 - 0.662
TYG-GGT	0.614	0.024	0.567 - 0.661
UACR	0.612	0.026	0.561 - 0.662
GGT	0.603	0.024	0.556 - 0.651
BMI	0.602	0.026	0.551 - 0.652
TyGIS	0.601	0.024	0.555 - 0.648
Age	0.591	0.027	0.539 - 0.643
AST	0.583	0.025	0.533 - 0.632
Hb	0.567	0.026	0.517 - 0.617
HOMA-IR	0.563	0.024	0.515 - 0.611
eGDR_BMI_	0.559	0.023	0.513 - 0.605
TC	0.519	0.024	0.529 - 0.567
LDL-c	0.495	0.025	0.544 - 0.553

BMI, body mass index; TC, total cholesterol; LDL-c, low-density lipoprotein cholesterol; TG, triglyceride; HDL-c, high-density lipoprotein cholesterol; ALT, alanine aminotransferase; AST, aspartate aminotransferase; GGT, gamma-glutamyl transferase; eGFR, estimated glomerular filtration rate; UACR, urinary albumin to creatinine ratio; Hb, hemoglobin; TyG–ALT, triglyceride–glucose–alanine aminotransferase index; HOMA-IR, homeostatic model assessment of insulin resistance; eGDR_BMI_, estimated glucose disposal rate; SPISE, the single point insulin sensitivity estimator; TyGIS, improved triglyceride glucose index; METS-IR, metabolic score for IR; log_e_ GDR, a natural log transformation of the glucose disposal rate.

### 3.5 Discussion

This cross-sectional study revealed a significant inverse correlation between log_e_ GDR and HUA prevalence. Our study revealed a significant log_e_ GDR decrease in the group with HUA, and HUA incidence declined progressively with increasing log_e_ GDR quartiles. Multivariable-adjusted regression models confirmed log_e_ GDR as an independent factor associated with HUA.

Our study revealed that log_e_ GDR was significantly associated with the aforementioned IR indices, with these markers decreasing progressively with increasing log_e_ GDR quartiles. Current research indicates a strong association between HUA and IR, with particularly prominent correlations observed with the triglyceride-glucose (TyG) index and TyG-BMI index (17). However, the correlation between log_e_ GDR and HUA has not been previously investigated. Here, we demonstrate an independent association between log_e_ GDR and HUA for the first time.

This study also incorporated other IR indices (TG/HDL-c ratio, TyG index, TyG-GGT, TyG-BMI, TyG-ALT, HOMA-IR, SPISE, TyGIS, METS-IR, and eGDR_BMI_) and common HUA-related markers (TC, HDL-c, and LDL-c) for comprehensive analysis. Only log_e_ GDR remained in the regression model, demonstrating its status as an independent factor associated with HUA. Area under the ROC curve analysis showed that log_e_ GDR outperformed other variables in HUA prediction in patients with T2DM. These results indicate that this composite index (log_e_ GDR) has significantly superior discriminative ability for HUA.

However, the mechanistic relationship between log_e_ GDR and HUA has not been elucidated. HUA is significantly associated with oxidative stress, MetS, and IR. HUA causes endothelial dysfunction via apoptosis, oxidative stress, and inflammation. However, it interferes with insulin signaling and decreases endothelial nitric oxide availability, resulting in endothelial IR (9), increased expression of urate transporter 1 (URAT1) and glucose transporter 9 (GLUT9), and glycolytic disturbances because of IR may be associated with HUA development in MetS (28). Log_e_ GDR includes the following key metabolic parameters: BMI, GGT, UACR, and TG, which are closely associated with HUA, oxidative stress, and MetS. A growing number of studies have shown a correlation between SUA and hypertriglyceridemia (HTG) (29). Studies have demonstrated a strong positive correlation between SUA and HTG (30). HTG is a core diagnostic criterion for MetS (31). Moreover, apolipoprotein E has been implicated in SUA-induced HTG (32). Apolipoprotein E4 leaves HDL more readily, enhancing the clearance of remnants, whose cholesterol downregulates hepatic LDL receptor expression, thereby increasing plasma LDL levels (33). This process elevates TG via the abovementioned mechanism. Additionally, recent evidence indicates that BMI is an important confounding factor in uric acid and metabolic disease research (34). Increased baseline BMI is significantly associated with higher HUA risk (35), which is partly attributable to obesity-induced IR, which enhances uric acid reabsorption in the proximal renal tubules while reducing uric acid and sodium excretion, leading to HUA (36, 37). However, IR cases have also been reported in individuals with a low BMI and a highly inflammatory state because of mast cell activation associated with very high oxidative stress. Mast cells produce α-melanocyte-stimulating hormone(α-MSH), a hormone that stimulates cortisol production, thereby increasing blood sugar. This causes excessive insulin production and, consequently, IR. Furthermore, GGT is significantly associated with HUA. Oxidative stress and MetS are related to HUA; GGT levels are also associated with MetS and oxidative stress (38). GGT’s physiological role of counteracting oxidative stress by breaking down extracellular glutathione and making its component amino acids available to cells makes it a potential oxidative stress marker (39). Studies show that UACR is significantly associated with increased uric acid. Uric acid was an independent factor for a 1-year increase of UACR [coefficient 4.80 (95% CI: 0.40–9.33) (mg/g) per 1-mg/dL increase in uric acid, P = 0.033] (40). This is probably because HUA plays a pathogenic role in chronic kidney disease development and progression by inducing inflammation, endothelial dysfunction, and activation of the renin–angiotensin system (41, 42). Furthermore, uric acid may increase oxidative stress, leading to mitochondrial dysfunction, proinflammatory cytokine oversecretion, and vascular smooth muscle cell proliferation, leading to renal function impairment (28). Additionally, unlike conventional IR indices, log_e_ GDR innovatively incorporates the UACR. Given the well-established association between UACR and HUA metabolism, combined with our demonstration that UACR is an independent risk factor for HUA, this might be the reason log_e_ GDR exhibits superior HUA prediction. In summary, all stratified log_e_ GDR subgroups demonstrate significant associations with key HUA metabolic pathways.

This study has limitations. Because of its cross-sectional design, it could establish an association between log_e_ GDR and HUA but not a causal relationship. Second, because this study was limited to patients with T2DM, it had a relatively small sample size of HUA cases. Future large-scale prospective studies are needed to further elucidate the relationship between IR and HUA. Moreover, the pathophysiological mechanisms underlying the association between log_e_ GDR and HUA require further investigation.

## 4 Conclusion

Log_e_ GDR may be a superior HUA predictor and an effective HUA marker in patients with T2DM. However, the underlying mechanisms require further investigation.

## Data Availability

The raw data supporting the conclusions of this article will be made available by the authors, without undue reservation.
